# ASSOCIATION BETWEEN ANXIETY ABOUT AGING AND AGEISM TOWARD OLDER ADULTS AMONG KOREAN DENTAL HYGIENE WORKFORCE

**DOI:** 10.1093/geroni/igad104.2694

**Published:** 2023-12-21

**Authors:** Jenny Kwon, Michael Hughes, Anh Vo

**Affiliations:** Miami University, Oxford, Ohio, United States; Miami University, Oxford, Ohio, United States; Miami University, Oxford, Ohio, United States

## Abstract

Ageism toward older people is prevalent in Korean healthcare settings, where older adults are likely to encounter other age groups of people. However, not many studies took close attention to ageism in the dental hygiene field. Considering the increase in dental care demand among the older population, this study aims to investigate the level of ageism and the factors associated with ageism among Korean dental hygiene undergraduates and dental hygienists. Based on the convenience sampling strategy, a total of 146 online surveys were collected from July to September 2022 in Seoul and Daejeon, Korea. Aging anxiety, ageist attitude, intergenerational contact measures, demographic characteristics, and geriatric-related experiences of participants were asked. Higher scores indicate lower aging anxiety and higher ageism. The mean (±SD) age of participants was 24 (±6.05) years. The mean score of aging anxiety was 2.97 (±.52) out of 5 and the mean score of ageism was 2.10 (±.31) out of 7. In the multiple regression model, aging anxiety is significantly associated with ageism (β = −0.257, p < 0.001). Participants feeling more anxious about aging have a more ageist attitude toward older people. The results suggest that well-designed gerontological education and intergenerational programs are necessary. In the short term, these efforts will decrease the fear of aging among dental hygiene field workers. In the long term, these will help them have the right understanding of aging and a positive attitude toward older people which can affect the quality of dental care service.

